# Cost-Cutting or Trust Building: Consumer Motive Inference and Purchase Intention Toward AI-Produced Food

**DOI:** 10.3390/foods15081405

**Published:** 2026-04-17

**Authors:** Chenhan Ruan, Yuanyuan Quan, Xu Li, Yi Zheng, Hengshan Deng, Xia Wei

**Affiliations:** 1 School of Economics and Management, Fujian Agriculture and Forestry University, Fuzhou 350002, China; chenhanruan@126.com (C.R.); q18779665554@163.com (Y.Q.); coratrump019@gmail.com (X.L.); quancora887@gmail.com (H.D.); 2 Green Value Chain Management Research Center, Fujian Agriculture and Forestry University, Fuzhou 350002, China; 3 School of Management, Shenzhen University, Shenzhen 518060, China; weixia@szu.edu.cn

**Keywords:** artificial intelligence, motive inference, food production, corporate reputation

## Abstract

Artificial intelligence (AI) has gradually been applied to food production. Many companies now face a choice between adopting AI technology and adhering to the traditional methods of food production. Existing studies have reported inconsistent findings regarding consumer perceptions of AI-produced food, yet little research has examined how consumers form motive inferences on businesses that transition from traditional practices to adopting AI in new food development. Based on motivation inference theory, this paper investigates the impact of food production methods on consumer inferences and purchase intention. Through three experiments, we find that AI-produced food evokes more negative motive inference in trust building and lowers purchase intention than traditionally produced food. Furthermore, such effect is driven by a serial mediating effect through cost-cutting attribution and perceived ulterior motive. Additionally, it is attenuated when the food company has a high corporate reputation. This research advances research on AI application in food from a consumer motive inference perspective, providing suggestions on firms’ adoption of AI-based practices in food production.

## 1. Introduction

In recent years, artificial intelligence (AI) has evolved from a futuristic concept to a practical, transformative force reshaping the entire food production chain, ranging from quality control to supply chain optimization [1,2]. AI applications in food production (e.g., AI farmer, AI chef, and AI barista), refer to the integration of artificial intelligence to automate food production processes, including the regulation of produce cultivation, receipt generation, food processing, new food development, and quality inspections [3,4]. It can control various factors like temperature, time, and humidity throughout produce growth and food processing and can even develop personalized taste plans based on consumer data [4,5]. According to Research Nester (2024), the global AI market size in the food and beverage industry is projected to surge from $14.41 billion in 2025 to $382.44 billion by 2035, representing a compound annual growth rate (CAGR) of 38.8% [6]. Major food enterprises such as Nestlé have adopted artificial intelligence to combine insights from consumers in new food development [7]. In addition, governments and regulatory authorities have also introduced corresponding policies governing AI applications in the food industry. For example, the Chinese government has implemented a series of policies to encourage AI adoption in food systems, including supply chain supervision, food market information analysis, and intelligent food cultivation [8]. The European Union AI Act imposes stringent requirements for transparency, safety validation, and ethical compliance to mitigate potential hazards associated with algorithmic decision-making in food systems [9].

Extant research has mainly emphasized the supply-side benefits of AI-driven food production [10,11], yet it lacks the consideration of consumer psychological reactions towards AI food. Some recent findings have revealed the negative perceptions towards AI technology in food production, such as negative concerns about safety, lower quality, and perceived lack of naturalness [12,13,14]. This is because consumers usually associate traditional production with human emotional involvement and trust [15,16], higher perceived authenticity [17,18], and higher quality due to concerns about cultural preservation [19]. However, such perceptions mainly stem from the “machine effect” of AI technology compared to the “human effect” and lacks the exploration of how consumers make inferences toward companies’ adoption of AI technology.

Drawing on motivation inference theory [20], this study posits that consumers tend to form negative inferences toward companies that adopt AI technology for food production. According to the theory, the shifting of production method serves as a critical cue to infer the true intentions behind the behavior [20]. Because consumers have a lay belief that enterprises are self-serving and profit-driven [21,22], AI adoption is likely to be perceived as a strategic attempt to reduce costs and increase profits [21,23]. This cost-cutting attribution would further lead to perceived ulterior motives, such as self-serving or opportunistic, at the expense of consumer welfare [24,25]. For instance, in service interactions, consumers tend to view the empathy expressed by AI robots as an insincere, mechanical display when its adoption is attributed solely to cost-cutting and a disregard for consumer welfare [26]. Likewise, in AI-driven checkout charity, when consumers perceive cost-reduction behaviors by retailers, they activate persuasion knowledge and attribute ulterior self-serving motives to the firms [27]. Therefore, across three experimental studies, we demonstrate consumers’ negative motivation inferences for AI versus the traditional production method. We further show that consumers perceive AI-adopting firms as primarily pursuing cost-cutting objectives, which triggers ulterior motive that dampens purchase intention. We also identify moderating factors such as corporate reputation in the above relationships.

This research contributes to AI application in the food domain from the perspective of consumer motivation inference. First, this research advances understanding of consumer perceptions towards AI-produced food, departing from the prominent AI’s positive effects from a technological advance perspective. Second, based on motivation inference theory, we further disclose a serial psychological path of cost-cutting attribution and perceived ulterior motives underlying the AI technology adoption. Third, we demonstrate variations of corporate reputation by considering moderating context of company-level characteristics. Practically, our findings highlight the need to systematically manage consumer inferences when adopting AI and to strategically consider a sustainable and consumer-centric production mode to build a positive brand image.

## 2. Literature Review and Hypotheses

### 2.1. AI Food and Consumer Reactions

Artificial intelligence is fundamentally redefining our interaction with technology and catalyzing a paradigm shift across sectors such as healthcare, finance, service, and food production [10,28]. Prior research has predominantly highlighted the positive impacts of AI-based food production from the supply-side perspective, such as increasing production yield and enhancing product quality [29]. Consumers generally perceive that AI technology can provide more reliable guarantees for food safety and health quality through precision management and real-time monitoring [10]. However, some recent studies have reported contradictory findings. For example, traditional production methods are familiar and have concern for cultural heritage [19], whereas AI production lies outside this common understanding [4]. Furthermore, consumers inherently associate food with nature, demonstrating a clear preference for naturally grown products and handmade items over those produced by machines [30,31]. Furthermore, consumers often view AI-made products as lacking warmth, as they believe that love and sincerity can only be transferred from human touch [32,33]. However, these perceptions are largely driven by the “machine effect” of AI, reflecting intuitive perceptions shaped by the technological nature of AI technology.

According to the motivation inference theory, we propose that consumers will make inferences about the motives behind the enterprises adopting AI technology. When enterprises disclose AI production signals, consumers will speculate on the motives of enterprises based on these signals. First, consumers have a lay belief that firms are profit-driven [34], believing that the behavior of enterprises often conflicts with social welfare and that they have self-serving motives such as cost reduction [22]. Traditional methods require investment in people, time, and experience, while AI reduces costs and increases efficiency [19], which makes consumers suspect that enterprises introduce AI not to optimize product quality but simply to cut costs. Rooted in the price–quality heuristic where consumers generally perceive higher prices as an indicator of superior quality [35], cost reduction is frequently equated with a sacrifice in product quality, and consumers tend to interpret this as enterprises being more concerned with cost savings rather than product quality optimization, thereby lowering their overall evaluation of the product. Additionally, as a product category carrying special emotional bonds, food is often perceived by consumers as being inseparable from human involvement and input in its production process [19]. The intervening of AI can easily be perceived as lacking kindness and warmth [13] or even regarded as an insincere and utilitarian technical approach that neglects humanistic values and emotional care. In contrast, traditional production methods are more likely to be perceived as sincere, attentive, and humanistic [13,19,32], thereby gaining greater consumer trust and recognition. Although the application of AI technology represents a form of corporate innovation, once consumers attribute its adoption to cost-cutting motives, this will offset the positive value of the technology, amplify inferences of negative motives, and ultimately lead to lower purchase intentions.

**H1.**  *Production method (AI vs. traditional) significantly influences consumers’ purchase intention. Specifically, AI production leads to lower purchase intention compared to traditional production methods.*

### 2.2. The Serial Mediating Effect of Cost-Cutting Attribution and Perceived Ulterior Motive

Motive inference theory refers to the process in which an individual (observer) in social interactions integrates the behavioral characteristics of the target object and the situational cues to make psychological inferences about the true intentions, purposes, or driving forces behind the behavior [20]. The core of this theory lies in distinguishing whether the behavior is driven by altruistic and pure motives (e.g., genuine concern or pursuit of social values) or self-interested and hidden motives (e.g., pursuit of personal or commercial interests) [36,37]. For instance, in the field of public welfare, consumers identify motives through entry sequence, viewing the early adopters as having pure motives and the followers as having self-interested motives [36]. They also see recommendations with material rewards with “hidden motives”, which undermines the recommendation effect and triggers negative social responses [38]. In human-robot interaction, people tend to think robots lack autonomous intent and thus make fewer motive inferences about them, making them more tolerant of robots’ insincere flattery than humans’ [39].

Production methods can often influence consumers’ motive inferences, leading them to judge the underlying true motives and thus affecting their evaluations of products and enterprises. In the context of this study, when enterprises disclose the use of AI technology in the research, development, and production of new products, due to consumers’ general lack of understanding of AI food products [10,40], they often infer the motives of enterprises based on their preconceived notions. This is because consumers have a lay belief that enterprises are driven by interests and are only profit-driven [21,22]. Cost-cutting can sometimes become the main action motives for companies to increase profits [21,23]. In the food industry, one prominent recognition of AI technology is that it can improve efficiency and reduce costs [41]. So consumers are likely to infer that companies shifting from traditional to AI technologies have self-interested motives such as cost reduction.

Moreover, when consumers perceive that firms adopt AI mainly to reduce costs, they will further assume that such firms hold hidden selfish or profit-driven motives [42]. This reflects the prevalent suspicion that enterprises prioritize their own economic interests in business decisions while neglecting the safeguarding of consumer value and satisfaction. On the one hand, the perceived cost-cutting directly triggers consumers’ inferences of corporate self-interest [36]. Consumers tend to equate enterprises’ cost-reduction behaviors with compromised product quality and diminished consumption experiences [24,25], assuming that such actions are motivated by a narrow focus on cost minimization rather than genuine efforts to optimize products and services. On the other hand, such motive inferences will foster negative perceptions of enterprises as insincere and irresponsible, as consumers believe that enterprises adopt AI technology to pursue technological shortcuts and follow industry trends for utilitarian goals. In contrast, traditional production is often viewed positively by consumers as pragmatic and responsible to both products standards and consumer well-being [19,43]. Therefore, when consumers attribute a food company’s adoption of AI production to cost-cutting, they will perceive the enterprise as having ulterior motives, thereby reducing their product evaluations and purchase intentions.

**H2.**  *The impact of food production methods (AI vs. traditional) on purchase intention is mediated by consumers’ cost-cutting attribution, which in turn leads to the perceived ulterior motive.*

### 2.3. The Moderating Effect of Corporate Reputation

According to Barnett et al. (2006), corporate reputation can be defined as the accumulated assessment of a firm’s financial, social, and environmental contributions over time [44]. These dimensions are critical in building consumer trust, encouraging behaviors such as adoption and continuance intention [45,46]. Organizations with higher reputations are perceived as more credible and socially responsible, thereby fostering more favorable interpretations of their underlying motives [47]. A firm’s historical investments lay the foundation for its corporate reputation, which then serves as a vital quality signal influencing how the market perceives the firm’s current quality [48]. Notably, in the food sector, corporate reputation similarly impacts how consumers evaluate food quality [35].

A firm’s high reputation exerts a positive influence on consumers’ attitudes toward its new products and their perceptions of the products’ attributes [49]. When disclosing the adoption of AI, high-reputation enterprises benefit from the halo effect [50], which reduces perceived risk and leads consumers to perceive AI-based production as a method for enhancing efficiency and quality stability. Furthermore, high reputation is typically associated with prosocial behavior [24,51,52]. Consumers tend to believe that high-reputation food enterprises adopt AI to enhance quality, optimize processes, ensure safety, and improve welfare. Therefore, these motives are largely attributed to efficiency gains or quality improvement [53,54]. Based on their trust in enterprises with high corporate reputation, consumers perceive AI usage as a commitment to efficiency or quality rather than mere cost reduction. In contrast, lower corporate reputations often translate to insufficient credibility, leading consumers to harbor negative perceptions and critically examine the intentions of enterprises’ AI-related initiatives. In this context, consumers are prone to dismiss AI adoption as a purely profit-driven maneuver, where product quality and consumer welfare are sacrificed for operational cost-cutting. Such negative framing inevitably reinforces inferences of ulterior motives and a singular focus on cost reduction [24,55], thereby leading consumers to attribute ulterior motives to the enterprise.

**H3.**  *Corporate reputation moderates the relationship between production method (AI vs. traditional) and purchase intention. Specifically, for firms with a high reputation, the negative effect of AI production on purchase intention is attenuated.*

**H4.**  *Corporate reputation moderates the relationship between the production method (AI vs. traditional) and the purchase intention via a serial psychological process of cost-cutting attribution and perceived ulterior motive.*

**Figure 1:**
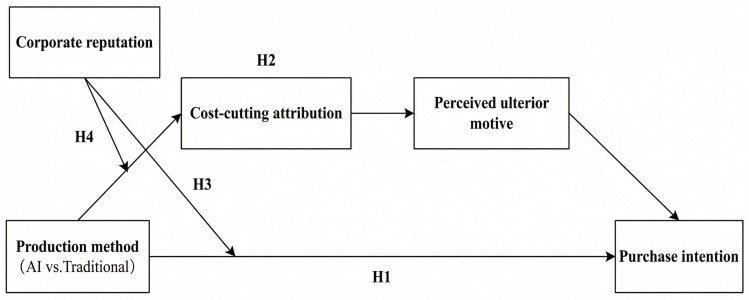
Research framework.

Overall, the proposed research framework is illustrated in Figure 1.

## 3. Methodology

### 3.1. Study Overview

We employed the experimental methods to test the hypotheses. All three studies adopted a scenario-based experimental design in which participants were presented with product labels or descriptions to explicitly indicating either AI-driven or traditional production methods. This approach is widely adopted for examining causal relationships among variables, as it enables the creation of controlled scenarios that minimize the influence of confounding factors. Study 1 was designed to verify the main effect, Study 2 aimed to test the serial mediating effect of cost-cutting attribution and perceived ulterior motives, and Study 3 was conducted to examine the moderating effect of corporate reputation along with the moderated mediation model.

To enhance the external generalizability of the findings, we selected different product categories as experimental stimuli across studies (please see Table 1 for more details). Notably, Fuding white tea, wine, and rice wine were chosen as experimental stimuli based on two key considerations. First, these categories represent traditional food and beverage categories with unique, heritage-based production methods that have long been rooted in human craftsmanship and manual operations. As a result, consumers hold strong expectations for the traditional production paradigms, and thus such products provide a conservative and rigorous test for AI-driven production. Second, these products are increasingly seeing real-world AI applications (e.g., AI-assisted fermentation, intelligent processing, automated quality control), making the context highly realistic and practically relevant.

Three distinct groups of participants were recruited online, with no overlap across studies. Informed consent was obtained online from all participants, and all studies received ethical approval from the Institutional Review Board of Fujian Agriculture and Forestry University. To ensure adequate statistical power, G * Power 3.1 was employed to calculate the minimum required sample size for each study [56]. Data analysis was performed using SPSS 26.0 (statistical package for the social sciences), a widely adopted tool in consumer behavior research.

### 3.2. Study 1: Testing the Main Effect

The purpose of Study 1 is to test the main effect of production methods (AI vs. traditional) on purchase intention. We expect that consumers demonstrate lower purchase intention on food produced by AI than similar food that is traditionally produced. Additionally, we controlled for several confounding variables to ensure the internal validity of our findings. First, we accounted for transferred essence, since traditional production is often perceived as a process through which a creator’s essence is infused into a product, thereby imbuing it with unique authenticity and value [32,57]. Second, we controlled for perceived naturalness to mitigate the common natural intervention by technology [58], Third, we controlled for perceived corporate social responsibility to ensure that consumer evaluations were not confounded by ethical concerns. Since AI-based production is frequently associated with the displacement, consumers might penalize AI-produced goods due to perceived unethical conduct [59], rather than the proposed cost-cutting motive attribution. Accounting for these variables allows us to isolate the unique psychological impact of the AI production method more accurately.

#### 3.2.1. Participants

Study 1 employed a factorial between-subjects design, with production method (AI vs. traditional) as the independent variable. To ensure adequate statistical power, we used G * Power 3.1 to determine the required sample size. Based on a medium effect size (Cohen’s f = 0.25) and a power of at least 0.80, the analysis indicated that a minimum total sample of 128 participants (64 per group) was required. Therefore, we recruited participants through Credamo (www.credamo.com), an online panel based in China. To ensure the integrity of the data, we excluded responses with abnormally long or short completion times and those that failed the embedded attention-check questions. This screening process ensured that only high-quality and attentive responses were retained for the final analysis. A total of 199 questionnaires were distributed, and 160 valid responses were retained for analysis. The sample consisted of 78.8% females with a mean age of 31.24 years (SD = 8.78). After signing the informed consent online, all participants completed the study and received a small monetary reward for their time.

#### 3.2.2. Measures

We randomly assigned participants to either the AI-driven or traditional production method group. Upon reviewing the instructions and passing an attention check, participants were informed that a Chinese white tea company was developing a new variety of Fuding white tea. They were then presented with an image of a box of Fuding white tea and a description. In the AI condition (N = 80), the packaging in the image was labeled “Produced via AI-based Methods”, and participants read a description of how Company A utilizes AI technology to produce the new food. Specifically, AI-based equipment and systems handle tea plucking, withering, fixation, rolling, fermentation and drying, relying on AI technology and data analysis. Conversely, in the traditional condition (N = 80), the packaging was labeled “Produced via Traditional Methods”, and the description emphasized Company A’s adherence to traditional production techniques. Specifically, skilled workers manually handle plucking, withering, fixation, rolling, fermentation and drying these tea processing steps, relying on their experience and craftsmanship (see Appendix A).

After that, participants rated their purchase intention, transferred essence, perceived naturalness, and corporate social responsibility. All variables were assessed using 7-point mature scale ranging from 1 (not at all) to 7 (very much). Specifically, purchase intention was measured using three items: “I will most certainly buy Fuding white tea from Company A in the future”; “There is a strong chance that I will buy Fuding white tea from Company A in the future”; “I will most likely buy Fuding white tea produced by Company A” (α = 0.87; adapted from Bianchi and Mortimer, 2015) [60]. Transferred essence was measured with the following statements: “The new food contains the true essence of white tea”; “The new food embodies the rich history and enduring traditions of white tea”; “The new food reflects the inheritance and classics of white tea” (α = 0.84; adapted from Newman and Dhar, 2014) [61]. Perceived naturalness was measured by asking such as to what extent the new food is “natural” (adapted from Hagen, 2021) [62]. Corporate social responsibility was measured via four items: “Company A actively helps to solve social problems through its business operations”; “Company A is concerned with improving the general well-being of society”; “Company A assumes a role in society that goes mere profit generation”; “Company A engages in philanthropy, contributing to causes such as the arts, education, and social services” (α = 0.87; adapted from Tiep et al., 2021) [63]. Finally, participants completed the manipulation check on their perceived production method by identifying which method Company A was stated to use in producing the new type of Fuding white tea (1 = traditional, 7 = AI). Finally, participants completed a brief demographic questionnaire regarding their age and gender.

#### 3.2.3. Results

In line with previous research [64,65], we employed dummy coding to test our predictions, assigning 1 to AI-based production and 0 to traditional production.

Manipulation Check. The results of the independent *t*-test indicated a significant difference in perceived production methods (M_AI_ = 6.69, SD_AI_ = 0.69; M_traditional_ = 1.20, SD_traditional_ = 0.75; t (158) = 48.18, *p* < 0.001). Furthermore, the two groups did not differ significantly in terms of transferred essence (M_AI_ = 5.76, SD_AI_ = 0.94, M_traditional_ = 5.99, SD_traditional_ = 0.80, t (158) = −1.69, *p* = 0.093), perceived naturalness (M_AI_ = 5.91, SD_AI_ = 0.97, M_traditional_ = 6.15, SD_traditional_ = 0.83, t (158) = −1.67, *p* = 0.098), and corporate social responsibility (M_AI_ = 5.09, SD_AI_ = 1.20, M_traditional_ = 5.21, SD_traditional_ = 0.98, t (158) = −0.70, *p* = 0.484). Therefore, the manipulation was successful.

Main Effect. An independent-samples *t*-test reveals that participants in the traditional condition demonstrate higher purchase intention (M_traditional_ = 5.87, SD_traditional_ = 0.80) than those in the AI condition (M_AI_ = 5.49, SD_AI_ = 1.15; t (158) = −2.40, *p* = 0.017). Thus, H1 is confirmed.

### 3.3. Study 2: The Sequential Mediating Effect

Study 2 aims to replicate the results of Study 1 (H1) and test the sequential mediating effect of cost-cutting attribution and perceived ulterior motive (H2) through a 2 (production method: AI vs. traditional) factorial between-subjects experimental design. Moreover, the stimulus in Study 2 was transitioned to wine, because the wine industry features well-defined and widely recognized traditional production standards, including manual harvesting and clay amphora fermentation [19], along with the increasing integration of artificial intelligence in modern winemaking [66].

#### 3.3.1. Participants

Participants were recruited via the Credamo website (www.credamo.com). To ensure data integrity, we implemented a rigorous screening process, excluding responses with abnormally long or short completion times and those that failed the embedded attention-check questions. Following this procedure, 200 valid responses were retained for further analysis. The final sample consisted of 74.5% females, with a mean age of 31.58 years (SD = 9.61). After signing the informed consent online, all participants completed the study and received a small monetary reward for their time. Additionally, all participants in this study were newly recruited and had not participated in Study 1.

#### 3.3.2. Measures

The manipulation of the production method was adopted from previous research [19]. Participants were shown a news report describing a new type of wine developed by Company A. The report provided a concise summary of the wine’s production process and the core principles underpinning its development. In the AI condition (N = 100), participants were informed that the new wine was produced using artificial intelligence, specifically through an intelligent algorithmic system that autonomously monitors and regulates the fermentation temperature via integrated digital sensors. In the traditional condition (N = 100), participants were instead informed that the new wine was produced through conventional methods; specifically, the winemaker conducted fermentation in clay amphorae, with temperature regulation achieved by positioning the vats upon blocks of ice (see Appendix B).

After that, we assessed purchase intentions, cost-cutting attributions, and perceived ulterior motives across both production scenarios. All variables were measured using mature scales on a 7-point Likert scale (1 = strongly disagree, 7 = strongly agree). Consistent with Study 1, purchase intention was assessed via a three-item: “I will most certainly buy new wine from Company A”; “There is a strong chance that I will buy new wine from Company A in the future”; “I will most likely buy new wine produced by Company A” (Cronbach’s α = 0.87; adapted from Bianchi and Mortimer, 2015) [60]. To assess cost-cutting attribution, participants responded to the following: “Company A wants to cut costs at the customers’ expense”; “Company A attempts to increase its profits by reducing labor costs in wine production”; “Company A prioritizes its own financial interests over the best interests of its customers” (Cronbach’s α = 0.84; adapted from Castelo et al., 2023) [21]. Perceived ulterior motive was measured using the following three items: “The intent of Company A is suspicious”; “The intent of Company A is insincere”; “Company A is attempting to inappropriately influence consumer decision-making” (Cronbach’s α = 0.93; adapted from Lee et al., 2023) [67]. Lastly, following the procedure in Study 1, participants completed a manipulation check by identifying the production method (1 = traditional, 7 = AI). Then we collected demographic information of participants, including age and gender.

#### 3.3.3. Results

Manipulation Check. The results of an independent-samples *t*-test show a significant difference in production method (M_AI_ = 6.63, SD_AI_ = 0.60, M_traditional_ = 1.19, SD_traditional_ = 0.42, t (198) = 74.56, *p* < 0.001). Therefore, the manipulation check is successful.

Main Effect. An independent-sample *t*-test shows that the traditional condition (M_traditional_ = 4.88, SD_traditional_ = 1.12) is more preferred than the AI condition (M_AI_ = 4.18, SD_AI_ = 1.55, t (198) = −3.66, *p* < 0.001) in purchase intention.

Sequential Mediating Effect. The results of independent-samples *t*-tests reveal that participants in the AI condition (M_AI_ = 3.88, SD_AI_ = 1.57) perceive significantly higher cost-cutting attributions than those in the traditional condition (M_traditional_ = 3.22, SD_traditional_ = 1.16, *p* = 0.001). Furthermore, participants in the AI condition (M_AI_ = 4.27, SD_AI_ = 1.69) perceive significantly higher perceived ulterior motive than those in the traditional condition (M_traditional_ = 3.50, SD_traditional_ = 1.51, *p* = 0.001). Next, we used Hayes’s (2017) PROCESS method to test the sequential mediating effects of cost-cutting attributions and perceived ulterior motive [68]. Using the production method as the independent variable, cost-cutting attribution as Mediator 1, perceived ulterior motive as Mediator 2, and purchase intention as the dependent variable, a sequential mediation analysis was conducted using Model 6 with a bootstrap sample size of 5000. The results indicate that the sequential effect of cost-cutting attribution and perceived ulterior motive is significant (indirect effect = −0.09, standard error = 0.04, 95% CI = [−0.1824, −0.0281]). This indicates that, when companies employ AI technology in new food development, consumers demonstrate stronger cost-cutting attribution, which fosters the perception of an ulterior motive of the company and ultimately reduces purchase intention. Thus, H2 is supported.

### 3.4. Study 3: The Moderating Effect of Corporate Reputation

Study 3 aims to replicate the findings of the prior studies and examine the moderating role of corporate reputation (H3). We further verify that this moderating effect is explained by the sequential mediation of cost-cutting attribution and perceived ulterior motive (H4). We also rule out potential explanations of functional value and hedonic value associated with food. Food produced by traditional techniques is commonly perceived to have better functional quality and can convey emotional value to consumers due to its association with cultural heritage and manual touch [13], which may further enhance food valuation.

#### 3.4.1. Participants

The sample size was determined using an F-test (ANCOVA: fixed effects, main effects, and interactions) conducted in G * Power 3.1, assuming a medium effect size (Cohen’ f = 0.25) and a statistical power of 0.80. This calculation yielded a minimum required sample size of 180 participants (45 per condition). Participants were recruited via Credamo (www.credamo.com), all of whom provided informed consent. Initially, 223 participants were randomly assigned to one of the four experimental conditions. After excluding those who failed the attention checks, a final sample of 200 valid responses was retained. These participants received a small monetary incentive for their time. The final sample consisted of 55.5% female participants, with a mean age of 31.32 years (SD = 8.56).

#### 3.4.2. Measures

Participants were informed that they were participating in a market research survey regarding the pre-market evaluation of a new rice wine product. Participants were presented with an image of a new rice wine from Company A. They were randomly assigned to one of four conditions in a 2 (production method: AI vs. traditional) × 2 (corporate reputation: high vs. low) between-subjects design. The manipulation of the production method was consistent with Study 2. The product description provided a concise summary of the rice wine’s production process. In the AI condition (N = 100), participants were informed that the rice wine is produced using advanced artificial intelligence systems. Specifically, the process involves utilizing AI-based sensors and algorithms to precisely monitor the fermentation of the grains, with the system automatically adjusting temperatures and environmental parameters in real-time to ensure optimal product consistency. In the traditional condition (N = 100), participants were informed that the winemaker employs time-honored methods; specifically, the process involves steaming the whole grains in wooden vats and allowing them to naturally ferment under the careful supervision of master brewers. The manipulation of corporate reputation was adapted from previous research [69]; participants were told that they had recently read a media report about Company A. In the high reputation scenario, the report cited a recent ranking of rice wine brands, which listed the company as “number one in taste and quality”. It then went on to describe an interview with its chief executive officer, who mentioned the company’s consistent rice wine quality and outstanding production efficiency, emphasized the firm’s strong financial background, and announced plans to expand the company’s scale. In the low reputation scenario, the report cited a current rice wine brand ranking which presented the company as “ranked last in taste and quality”. It went on to describe an interview with the chief executive officer, who stated that the company was facing economic difficulties and was unable to focus on improving the quality of its rice wine and production efficiency and announced mass lay-offs (see Appendix C).

After that, we measured participants’ purchase intention, cost-cutting attributions, perceived ulterior motive, functional value, and emotional value. All variables were rated using a 7-point Likert scale (1 = not at all, 7 = very much). Participants reported their purchase intention (Cronbach’s α = 0.91, adapted from Bianchi and Mortimer, 2015) [60], cost-cutting attributions (Cronbach’s α = 0.88, adapted from Castelo et al., 2023), and perceived ulterior motive (Cronbach’s α = 0.91, adapted from Lee et al., 2023), consistent with Study 2 [21,67]. Functional value was measured using a six-item scale including: “To what extent do you think the new rice wine from Company A is reliable”; “To what extent do you believe it is valuable”; “To what extent do you feel it functions well”; “To what extent do you think it fulfills your needs well”; “To what extent do you perceive it offers consistent quality”; and “To what extent do you perceive it performs well” (Cronbach’s α = 0.94, adapted from Belanche et al., 2021) [42]. Emotional value was measured through six items: “To what extent do you think the new rice wine from Company A is interesting”; “To what extent do you think it is enjoyable”; “To what extent do you think it makes you feel relaxed”; “To what extent do you think it makes you feel good”; “To what extent do you think it gives you pleasure”; and “To what extent do you think it makes you want to use it” (Cronbach’s α = 0.94, adapted from Belanche et al., 2021) [42].

Finally, we delivered the manipulation checks. The manipulation check of the production method is similar to prior studies. The manipulation check of corporate reputation was conducted using a three-item measure: “To what extent do you have a positive overall image of Company A”; “To what extent do you admire and respect Company A”; and “To what extent does Company A have a good overall reputation” (Cronbach’s α = 0.93, adapted from Riordan et al., 1997) [70]. Furthermore, the demographic information of participants was collected, including age and gender.

#### 3.4.3. Results

Manipulation Check. The results of an independent-samples *t*-test show a significant difference in production method (M_AI_ = 6.75, SD_AI_ = 0.44, M_traditional_ = 1.26, SD_traditional_ = 0.71, t (198) = 66.25, *p* < 0.001), as well as in corporate reputation (M_high_ = 5.83, SD_high_ = 0.71, M_low_ = 3.52, SD_low_ = 1.48; t (198) = 14.02, *p* < 0.001) between the two groups. Additionally, the confounding effects are not significant, including functional value (M_AI_ = 5.01, SD_AI_ = 1.10, M_traditional_ = 5.28, SD_traditional_ = 1.32, t (198) = −1.57, *p* = 0.118) and emotional value (M_AI_ = 4.82, SD_AI_ = 1.23, M_traditional_ = 4.97, SD_traditional_ = 1.40, t (198) = −0.81, *p* = 0.421). Therefore, the manipulation checks are successful.

Main effect. An independent-samples *t*-test revealed that the traditional condition (M_traditional_ = 5.21, SD_traditional_ = 1.03) was significantly more preferred than the AI condition (M_AI_ = 4.76, SD_AI_ = 1.29, t (198) = −2.72, *p* = 0.007) in purchase intention.

Sequential mediating effect. We conducted a sequential mediation analysis by using Hayes’s (2017) [68] PROCESS method (Model 6, bootstrap sample = 5000) to test the sequential mediating effects of cost-cutting attributions and perceived ulterior motive. Specifically, when the production method (AI vs. traditional) was the independent variable, purchase intention was the dependent variable and the cost-cutting attributions and perceived ulterior motive were the mediating variables, the indirect effect of cost-cutting attributions, and perceived ulterior motive (indirect effect = −0.12, standard error = 0.06, 95% CI = [−0.2532, −0.0267]). This indicates that cost-cutting attributions and perceived ulterior motive play sequential mediating roles in the effect of the production method on purchase intention. Thus, H2 was established.

**Figure 2:**
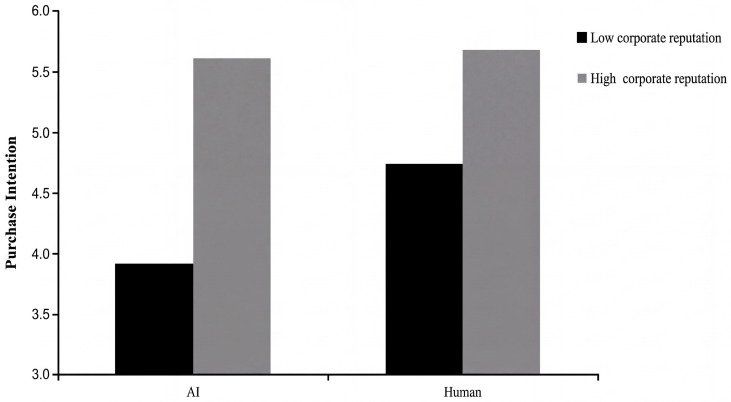
The moderating effect of corporate reputation.

Moderating effect of corporate reputation. We conducted a moderating analysis using the PROCESS macro Model 1 [68], with a bootstrap sample of 5000, production method as the independent variable, corporate reputation as the moderator, and purchase intention as the dependent variable. The 95% confidence interval for the moderating effect did not include zero, confirming that corporate reputation moderates the relationship between production method and purchase intention (effect = 0.77, standard error = 0.27, 95% CI = [0.2382, 1.2951], *p* = 0.005). Simple slope analysis reveals a significant main effect for participants who perceived low corporate reputation (effect = −0.83, standard error = 0.19, 95% CI = [−1.2070, −0.4597]), whereas the effect is non-significant for those who perceived high corporate reputation (effect = −0.07, standard error = 0.19, 95% CI = [−0.4403, 0.3070]; see Figure 2). This indicates that, when corporate reputation is high, there is no significant difference in purchase intention between AI production and traditional production. Therefore, the moderating effect of corporate reputation proposed in H3 is supported.

The moderated mediating effect. We conducted a moderated mediation analysis using the PROCESS macro, Model 83, with a bootstrap sample of 5000 [68], in which the production method was the independent variable, cost-cutting attributions and perceived ulterior motive were the sequential mediators, corporate reputation was the moderator, and purchase intention was the dependent variable. First, the results show that corporate reputation moderates the sequential mediating effects of cost-cutting attributions and perceived ulterior motive (effect = 0.16, standard error = 0.08, 95% CI = [0.0351, 0.3473]). This index of moderated sequential mediation is significant as the 95% confidence interval does not straddle zero. Specifically, under the condition of low corporate reputation, the production method had a significant impact on purchase intention through the serial mediation of cost-cutting attributions and perceived ulterior motive (effect = −0.20, standard error = 0.08, 95% CI = [−0.3779, −0.0740]). However, this indirect path was not significant when corporate reputation was high (effect = −0.04, standard error = 0.04, 95% CI = [−0.1282, 0.0302]). Thus, H4 is supported.

## 4. Discussion

### 4.1. Theoretical Contributions

Firstly, previous studies have primarily focused on the technological advance of AI applications in food production, such as improved efficiency, cost reduction, and technological innovation [10,29,71]. However, the existing literature overlooks the potential negative associations that AI may trigger among consumers. Although some recent studies have pointed out that consumers may hold negative associations toward AI-generated food, such as concerns about unnaturalness, lack of human emotions, and lack of uniqueness consideration [4,12], these studies have mainly focused on the perceptions that stem from the “machine effect” of AI technology [72,73]. However, studies have largely overlooked consumers’ inferences about the underlying motives of companies when adopting AI in new food development.

Secondly, grounded in motivational inference theory, this study reveals the serial mediating mechanism by which AI production affects consumer responses. Specifically, consumers may perceive AI adoption as an act of cost-cutting, which further triggers ulterior motive inference. Given that firms are often viewed as self-interested and profit-driven entities [36,74], consumers’ negative motivational inferences that the enterprises neglect consumer welfare may outweigh the positive ones in shaping their skepticism toward companies with AI adoption. Through a sequential mediation approach, it extends the application of motivational inference theory to AI food context to reveal the underlying psychological mechanisms in AI-produced food consumption.

Thirdly, previous studies on boundary conditions have mainly focused on consumer-level and product-level factors but has largely overlooked firm-level characteristics. For example, they have examined the moderating variables such as consumer characteristics (e.g., generation, product knowledge) and product characteristics (e.g., service type, packaging format) [75,76,77,78] but have paid less attention to the potential boundary effects of firm-level characteristics. This study identifies corporate reputation as a key moderating variable.

### 4.2. Managerial Implications

First, this study indicates that consumers demonstrate negative evaluations when companies choose to shift from traditional methods to AI methods in new food development. This suggests that the application of AI may not immediately gain consumers’ approval and favor. On the one hand, food enterprises can communicate more meticulously with consumers to convey the true value behind the application of AI technology and the benefits it brings, such as promoting product quality and ensuring food safety, thereby alleviating consumers’ concerns. On the other hand, manual production methods are still irreplaceable in some cases. Food enterprises can integrate traditional craftsmanship with innovative AI production models to meet the diverse needs of consumers. Policymakers can support this transition by issuing clear guidelines for responsible AI adoption in food production to reduce consumer uncertainty.

In addition, our findings highlight the critical importance for AI food firms to avoid triggering consumers’ negative motive inferences such as cost-cutting attribution and ulterior motive inference. Therefore, firms should avoid overemphasizing cost-oriented benefits and efficiency gains when introducing new technologies, as this may lead consumers to infer opportunistic and self-serving motives. Instead, managers should proactively communicate the value-added intentions behind their strategies, such as enhancing product safety, improving taste and nutritional value, or pursuing long-term sustainable development. To further guide corporate behavior, regulators can develop disclosure frameworks that encourage firms to highlight consumer-centric benefits of AI in food production.

Furthermore, corporate reputation plays a crucial role in consumers’ decisions to purchase AI-made agricultural products. The reputation of a company attenuates the negative inferences when companies adopt AI in new food development. Therefore, when promoting technological innovation and cost control, enterprises must pay attention to building their brand image and corporate reputation. By providing high-quality food, offering comprehensive after-sales services, and actively participating in social welfare activities, enterprises can effectively enhance their reputation and establish a good corporate image in the minds of consumers, thereby promoting consumer acceptance for AI technology in food application. Complementarily, policymakers can establish reputation-enhancing incentives, such as sustainability labels or safety accreditation, to promote the social image of some trustworthy AI-adopting enterprises.

### 4.3. Limitations and Future Directions

This research has several limitations that point to directions for future research. First, this research mainly adopts the experimental method for hypothesis testing. The external applicability of the findings can be strengthened by adding other research methods (e.g., secondary data and field data). Second, this paper mainly collects self-reported data from China, and for consumers from different cultures, their acceptance of artificial intelligence may vary [79]. Third, future research can explore more moderating variables such as food type. For instance, for functional foods, consumers may have a higher demand for precise control of nutritional components, and the precise and intelligent production capabilities based on AI are more likely to be valued by consumers. However, for hedonic foods, consumers pursue emotional experiences and demonstrate higher preference for foods with traditional inherited craftsmanship. Furthermore, future studies could investigate the moderating role of regulatory stringency or institutional transparency in AI food governance, which may mitigate consumers’ negative inferences toward AI production methods.

## 5. Conclusions

Across three studies, we demonstrate that AI-based food production reduces purchase intention compared to traditionally produced food (Study 1). Furthermore, drawing on motive inference theory, we show that this effect operates through a sequential mediation mechanism, in which AI-produced food triggers stronger cost-cutting attribution, which in turn fosters perceptions of ulterior motives (Study 2). Finally, we demonstrate that these effects are moderated by corporate reputation (Study 3). Specifically, for firms with a strong corporate reputation, consumers exhibit no significant difference in their purchase intentions between AI-produced and traditionally produced food. Conversely, when corporate reputation is weak, consumers report significantly lower purchase intention toward AI-produced food compared to traditionally produced food.

## Figures and Tables

**Table 1:** Study overview.

Study	Product	Purpose	Measures	Results
Study 1 (N = 160)	Fuding White Tea	Testing the main effect (H1).	Purchase intention.	Purchase intention: M_AI_ = 5.49, SD_AI_ = 1.15, M_traditional_ = 5.87, SD_traditional_ = 0.80, t (158) = −2.40, *p* = 0.017.
Study 2 (N = 200)	Wine	Testing the main effect (H1) and the sequential mediating effect (H2).	Purchase intention. Cost-cutting attribution. Perceived ulterior motive.	Purchase intention: M_AI_ = 4.18, SD_AI_ = 1.55, M_traditional_ = 4.88, SD_traditional_ = 1.12, t (198) = −3.66, *p* < 0.001; Sequential mediating Effect: Indirect Effect = −0.09, Standard Error = 0.04, 95% CI = [−0.1824, −0.0281]
Study3 (N = 200)	Rice wine	Testing the main effect (H1), the sequential mediating effect (H2), the moderating effect (H3) and the moderated meditating effect (H4).	Purchase intention. Cost-cutting attribution. Perceived ulterior motive.	Purchase intention: M_AI_ = 4.76, SD_AI_ = 1.29, M_traditional_ = 5.21, SD_traditional_ = 1.03, t (198) = −2.72, *p* = 0.007; Sequential mediating Effect: Indirect Effect = −0.12, Standard Error = 0.06, 95% CI = [−0.2532, −0.0267]; Moderating effect: Effect = 0.77, Standard Error = 0.27, *p* = 0.005, 95% CI = [0.2382, 1.2951]; Moderated meditating effect: Effect = 0.16, Standard Error = 0.08, 95% CI = [0.0351, 0.3473]

**Figure A1:**
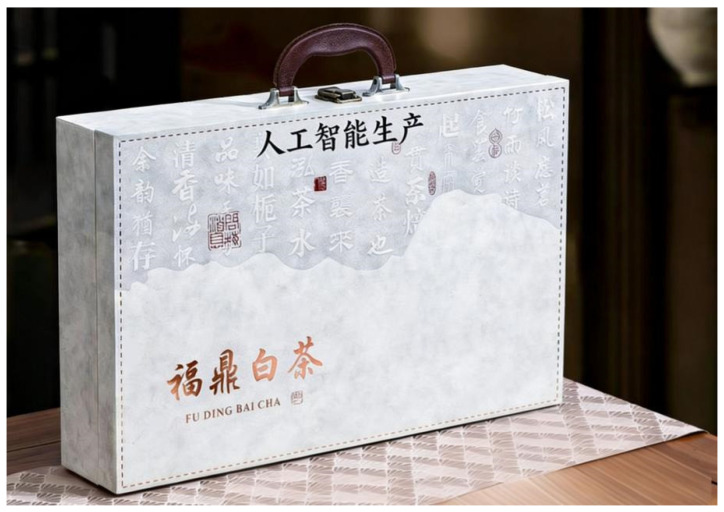
Manipulation stimuli picture for AI-Produced Fuding White Tea. **Note:** The Chinese text in the figure refers to AI-produced Fuding White Tea.

**Figure A2:**
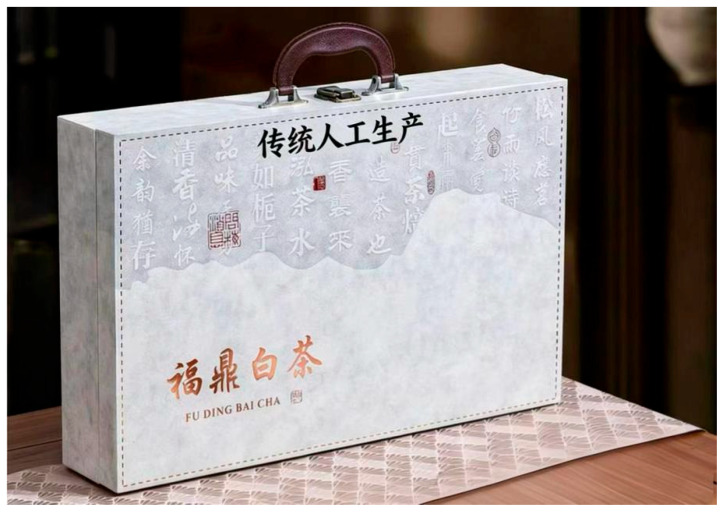
Manipulation stimuli picture for traditionally produced Fuding White Tea. **Note:** The Chinese text in the figure refers to traditionally produced Fuding White Tea.

**Figure A3:**
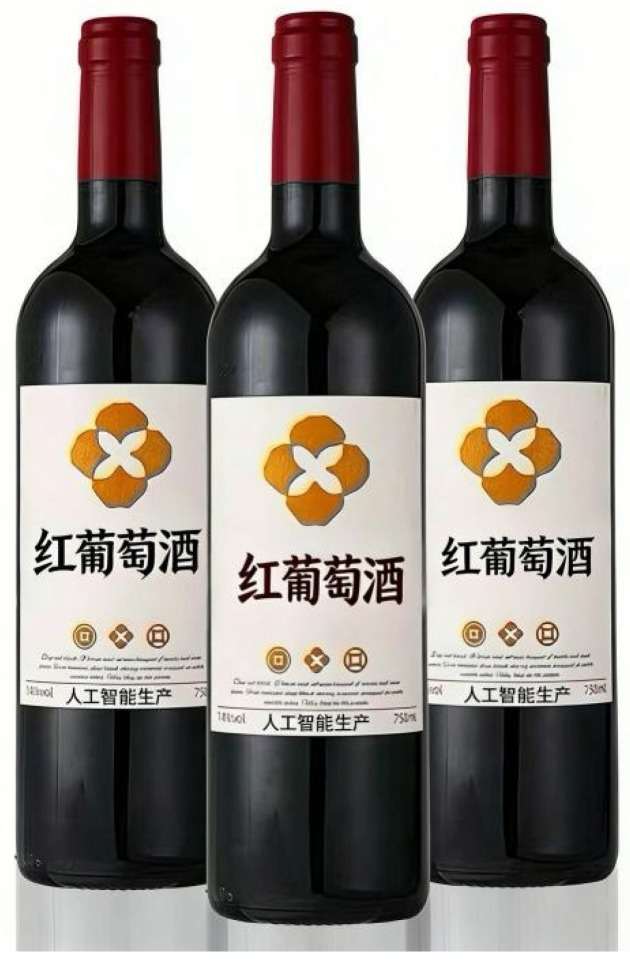
Manipulation stimuli picture for AI-produced red wine. **Note:** The Chinese text in the figure refers to AI-produced red wine.

**Figure A4:**
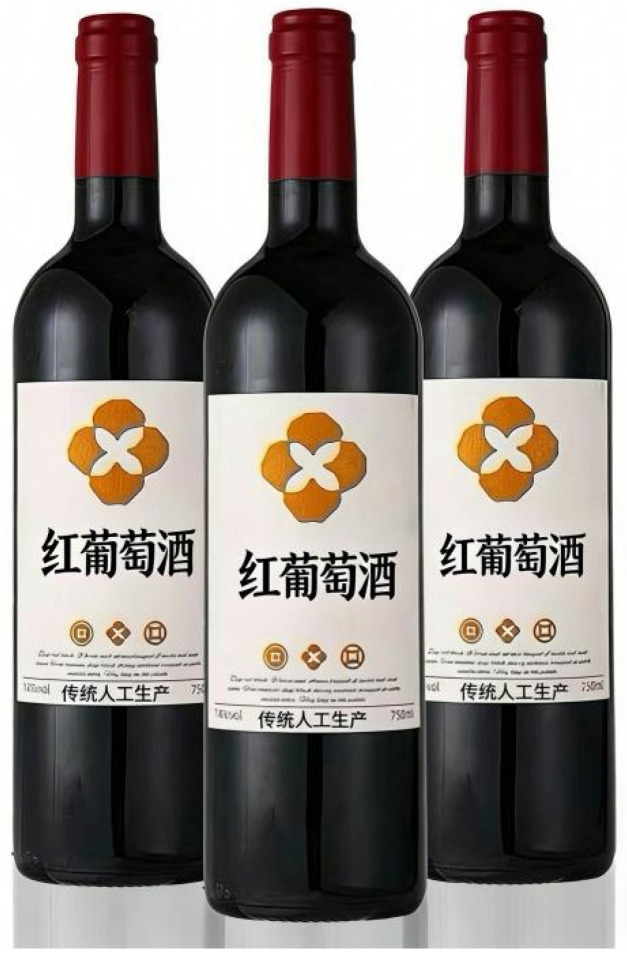
Manipulation stimuli picture for traditionally produced red wine. **Note:** The Chinese text in the figure refers to traditionally produced red wine.

**Figure A5:**
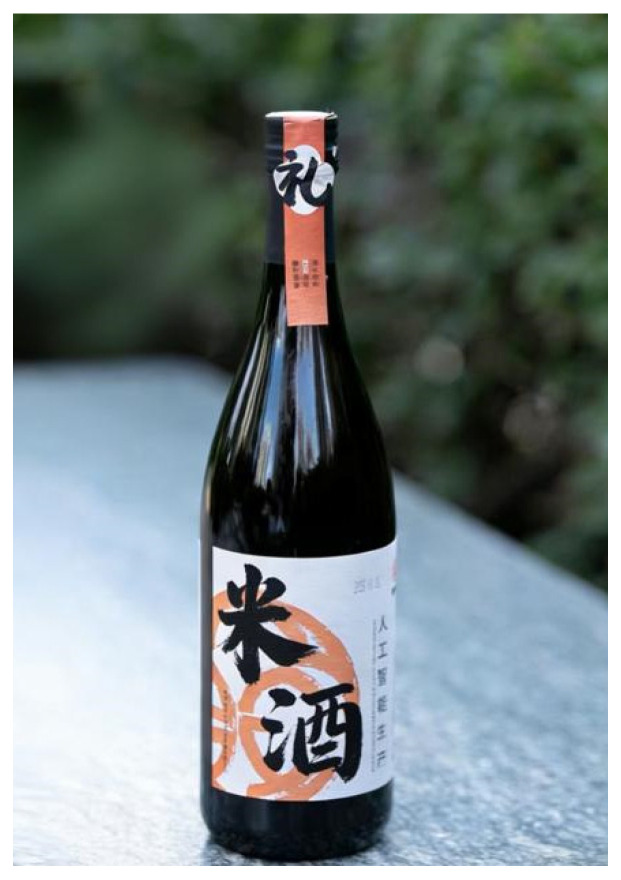
Manipulation stimuli picture for AI-produced rice wine. **Note:** The Chinese text in the figure refers to AI-produced rice wine.

**Figure A6:**
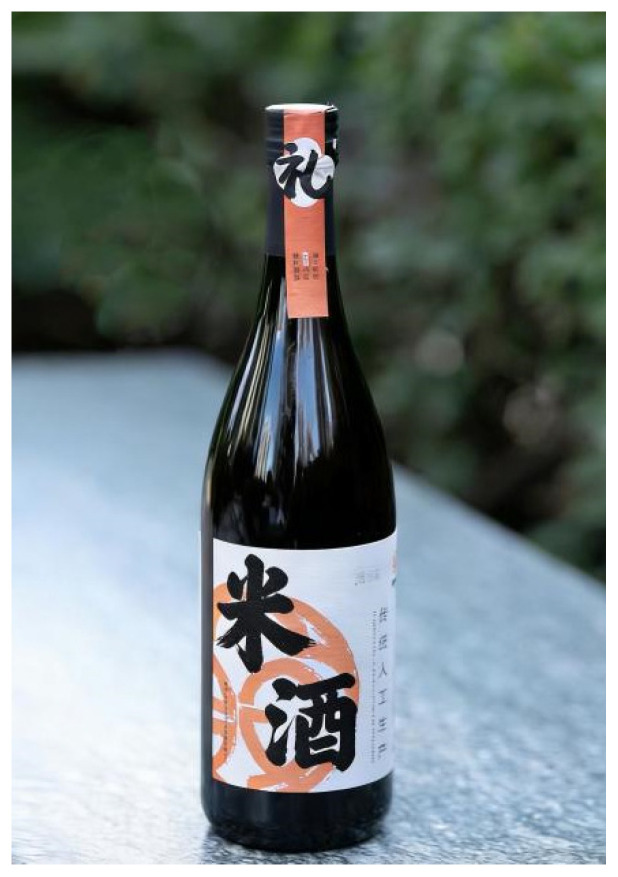
Manipulation stimuli picture for traditionally produced rice wine. **Note:** The Chinese text in the figure refers to traditionally produced rice wine.

## Data Availability

The original contributions presented in this study are included in the article. Further inquiries can be directed to the corresponding author.

## Appendix A. Manipulation Stimulus in Study 1

AI condition:

Company A uses artificial intelligence technology for tea production. AI-based robotic arms automatically pluck fresh tea leaves by preset standards, then place them in intelligent withering chambers with the AI system, precisely controlling temperature, humidity, and air flow. Next, AI-based fixation equipment adjusts heating parameters to inhibit enzymatic activity, and AI-driven rolling machines shape leaves and squeeze out juice. During fermentation, the AI system monitors and adjusts temperature, humidity, and time via sensors and machine learning to ensure consistent quality. Finally, tea leaves are dried or baked by AI equipment, with the whole process relying on AI technology, data analysis, and intelligent equipment.

Traditional Condition:

Company A adopts traditional manual tea processing techniques. Skilled workers manually pluck fresh tea leaves, then spread them naturally for withering to soften the leaves and reduce moisture. Next, workers carry out fixation to stop enzymatic activity, followed by hand-guided rolling to shape the tea leaves and release juice. For fermented teas, workers control the fermentation process entirely by experience according to ambient temperature and humidity. Finally, the tea leaves are dried or baked using traditional methods, before being manually sorted and graded to remove impurities. The whole process depends on craftsmanship, sensory judgment, and manual operation.

## Appendix B. Manipulation Stimulus in Study 2

AI condition:

The new wine from Company A is produced via an AI-based fermentation method. This process consists of fermenting a vat of grape juice and controlling the heat generated during fermentation through an intelligent algorithmic system that autonomously monitors and regulates the temperature.

Traditional Condition:

The new wine from Company A is produced by the local winemaker using a traditional fermentation method. This process consists of fermenting a vat of grape juice and controlling the heat generated during fermentation by placing the vat in a clay pot atop blocks of ice.

## Appendix C. Manipulation Stimulus in Study 3

AI condition:

The new rice wine from Company A is produced by advanced artificial intelligence systems, with the process including the utilization of AI-based sensors and algorithms to precisely monitor the fermentation of the grains, and the system automatically adjusts temperatures and environmental parameters in real-time based on the monitored data to ensure optimal product consistency in flavor, alcohol content, and texture.

Traditional Condition:

The new rice wine from Company A is produced using time-honored traditional craftsmanship, with the process involving steaming whole grains in wooden vats and allowing them to undergo natural fermentation under the supervision of master brewers, who rely on their experience to adjust fermentation conditions and ensure the product’s authentic flavor.
